# Nanobody‐mediated resistance to Grapevine fanleaf virus in plants

**DOI:** 10.1111/pbi.12819

**Published:** 2017-10-06

**Authors:** Caroline Hemmer, Samia Djennane, Léa Ackerer, Kamal Hleibieh, Aurélie Marmonier, Sophie Gersch, Shahinez Garcia, Emmanuelle Vigne, Véronique Komar, Mireille Perrin, Claude Gertz, Lorène Belval, François Berthold, Baptiste Monsion, Corinne Schmitt‐Keichinger, Olivier Lemaire, Bernard Lorber, Carlos Gutiérrez, Serge Muyldermans, Gérard Demangeat, Christophe Ritzenthaler

**Affiliations:** ^1^ Institut de biologie moléculaire des plantes du CNRS Université de Strasbourg Strasbourg France; ^2^ SVQV INRA Université de Strasbourg Colmar France; ^3^ Institut français de la vigne et du vin Domaine de l'Espiguette Le Grau du Roi France; ^4^ Institut de biologie moléculaire et cellulaire du CNRS Strasbourg Cedex France; ^5^ Research Institute of Biomedical and Health Sciences University of Las Palmas de Gran Canaria Arucas Las Palmas Spain; ^6^ Cellular and Molecular Immunology Vrije Universiteit Brussel Brussels Belgium

**Keywords:** nanobodies, plant virus, transgenic plant, grapevine, nepovirus, single‐chain antibodies, GMO

## Abstract

Since their discovery, single‐domain antigen‐binding fragments of camelid‐derived heavy‐chain‐only antibodies, also known as nanobodies (Nbs), have proven to be of outstanding interest as therapeutics against human diseases and pathogens including viruses, but their use against phytopathogens remains limited. Many plant viruses including *Grapevine fanleaf virus* (GFLV), a nematode‐transmitted icosahedral virus and causal agent of fanleaf degenerative disease, have worldwide distribution and huge burden on crop yields representing billions of US dollars of losses annually, yet solutions to combat these viruses are often limited or inefficient. Here, we identified a Nb specific to GFLV that confers strong resistance to GFLV upon stable expression in the model plant *Nicotiana benthamiana* and also in grapevine rootstock, the natural host of the virus. We showed that resistance was effective against a broad range of GFLV isolates independently of the inoculation method including upon nematode transmission but not against its close relative, *Arabis mosaic virus*. We also demonstrated that virus neutralization occurs at an early step of the virus life cycle, prior to cell‐to‐cell movement. Our findings will not only be instrumental to confer resistance to GFLV in grapevine, but more generally they pave the way for the generation of novel antiviral strategies in plants based on Nbs.

## Introduction

With well over 60 different viruses identified, grapevine (*Vitis* spp) is the crop with the highest number of infecting viruses (Martelli, 2014). Although the pathogenicity of all these viruses has not been established, a number of them are considered as severe grapevine pathogens such as the emerging *Red blotch virus* or *Grapevine pinot gris virus* or the well‐described viruses responsible for rugose wood, leafroll and fanleaf degenerative diseases (Basso *et al*., 2017; Maliogka *et al*., 2015). The latter is often considered to be the most detrimental and widespread grapevine viral disease as it affects vineyards worldwide, in particular those of high‐added value in which grapevine has been cultivated for centuries (Basso *et al*., 2017). Fanleaf degenerative disease is characterized by a range of symptoms that include yellow mottling and distortion of the leaves that can resemble a fan, malformed canes with exceedingly short internodes, smaller than normal clusters and overall stunted vines of reduced vigour (Schmitt‐Keichinger *et al*., 2017).

*Grapevine fanleaf virus* (GFLV) and to a lesser extent *Arabis mosaic virus* (ArMV) are the major causal agents of fanleaf degenerative disease. As members of the genus *Nepovirus* within the family *Secoviridae,* these viruses are transmitted in nature by ectoparasitic dagger nematode vectors of the genus *Xiphinema* that primarily feed on root tips (Andret‐Link *et al*., 2017). GFLV and ArMV possess a bipartite positive‐strand RNA genome. Their icosahedral capsid with *T *= pseudo3 symmetry is composed of 60 copies of the 54 kDa coat protein (CP) that plays essential functions in transmission by nematodes (Lai‐Kee‐Him *et al*., 2013; Schellenberger *et al*., 2010, 2011b).

Breeding is commonly used and remains the most efficient and practical way for the effective management of diseases in cultivated plants. In this manner, resistance to *Plasmopara viticola*, the causal agent of downy mildew, or to phylloxera has been widely deployed in grapevine (Collinge *et al*., 2010). However, so far, plant breeding has remained ineffective against grapevine viruses due to the absence of known sources of viral resistance in the *Vitis* germplasm (Oliver and Fuchs, 2011). As an alternative, genetic engineering has served to incorporate resistance to grapevine viruses and various strategies have been employed including pathogen‐derived resistance, RNA‐mediated resistance and plantibodies (Gottula and Fuchs, 2009; Laimer *et al*., 2009; Safarnejad *et al*., 2011; Simon‐Mateo and Garcia, 2011). The full spectrum of these strategies has been deployed for GFLV (Gambino *et al*., 2010; Jardak‐Jamoussi *et al*., 2009; Jelly *et al*., 2012; Krastanova *et al*., 1995; Mauro *et al*., 1995; Nölke *et al*., 2009; Vigne *et al*., 2004b; Xue *et al*., 1999), but their efficacy has only rarely been addressed in grapevine. Probably the most advanced study is provided by Vigne *et al*. (2004b) with transgenic grapevine rootstocks expressing the CP gene of GFLV, but resistance observed was insufficient for commercial use.

Recently, Ghannam *et al*. (2015) reported about nanobodies (Nbs) derived from heavy‐chain‐only antibodies found in camelids (Muyldermans, 2013) to confer resistance against viruses. More specifically, they showed that Nbs directed against *Broad bean mottle virus* (BBMV) displayed neutralizing activity against the cognate virus upon transient expression in *Vicia faba,* suggesting that Nbs could represent promising tools to immunomodulate plant resistance against viruses. Here, we report about the identification of a Nb specific to GFLV able to confer strong resistance to GFLV upon stable expression in both *Nicotiana benthamiana* and grapevine. We demonstrate that resistance is effective against a wide range of GFLV isolates but not against ArMV and is due to the neutralization of the virus at the initial stage of the virus life cycle, prior to cell‐to‐cell movement.

## Results

### Nb23 recognizes a broad range of GFLV isolates but not ArMV

**Figure 1:**
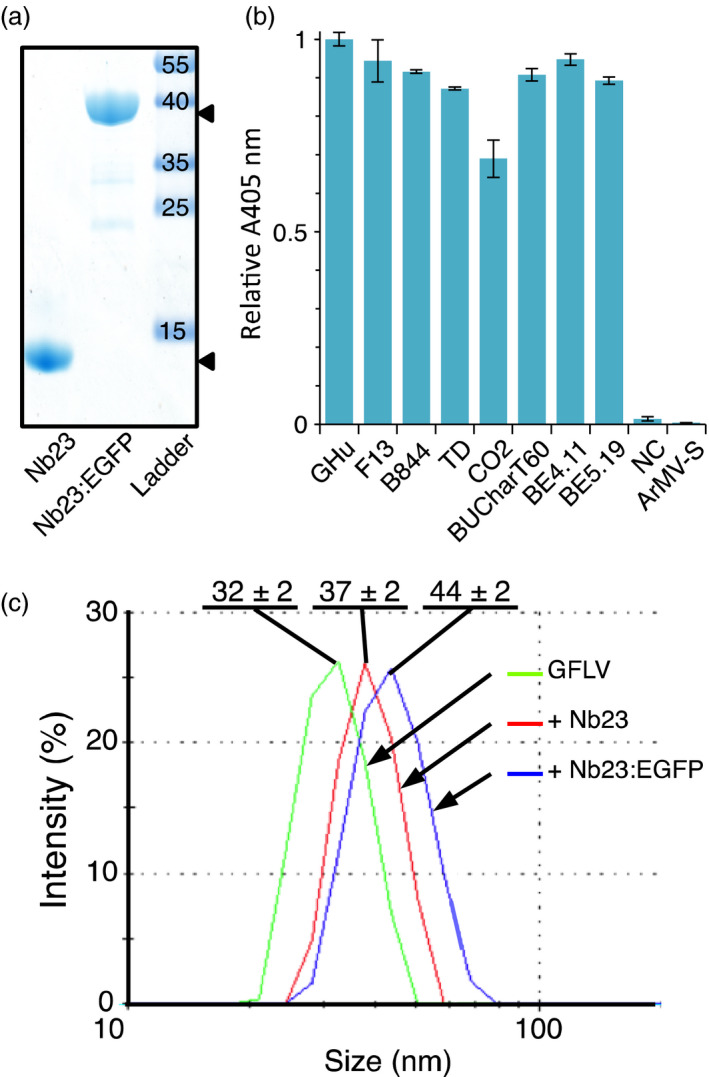
Nb23 recognizes different GFLV isolates but not ArMV and remains functional upon fusion to GFP. (a) Coomassie blue‐stained denaturing gel showing purified Nb23, Nb23:EGFP (arrowheads) and protein ladder with sizes (kDa). (b) Nb23‐based DAS‐ELISA results of *Chenopodium quinoa* infected with eight different GFLV isolates and ArMV‐S. NC, negative control (healthy plant). Values are means ± SD (*n *=* *2, experimental duplicates). (c) Dynamic light scattering (DLS) analyses of GFLV‐F13 alone (green curve) and GFLV complexed to Nb23 (red curve) or to Nb23:EGFP (blue curve). All particles were monodisperse with diameters of 32 ± 2 nm (mean ± SD,* n *=* *3) for GFLV, 37 ± 2 nm (mean ± SD,* n *=* *3) for GFLV saturated with Nb23, and 44 ± 2 nm (mean ± SD,* n *=* *3) for GFLV saturated with Nb23:EGFP.

Initially, 23 different Nbs belonging to 11 distinct families were isolated from an immune library generated against purified GFLV particles. Nb23 from this collection, which belongs to the family 1 with the highest number of representatives (Figure S1), was purified (Figure 1a) and found by double‐antibody sandwich enzyme‐linked immunosorbent assay (DAS‐ELISA) to recognize eight GFLV isolates (Figure 1b) differing in CP composition (Figure S2). Despite approximately 70% identity at the amino acid level between CPs, extensive structural similarities between viruses (Lai‐Kee‐Him *et al*., 2013) and the existence of GFLV‐ArMV heterospecific monoclonal antibodies (Frison and Stace‐Smith, 1992; Nölke *et al*., 2009), Nb23 failed to recognize ArMV isolate‐S (ArMV‐S, Figure 1b) in DAS‐ELISA. Similarly to Nb23, we managed to produce and purify Nb23 fused to EGFP (Nb23:EGFP) from *E. coli* that migrated in SDS‐PAGE at the expected MW of 39 kDa (calculated MW for Nb23:EGFP: 41.3 kDa) (Figure 1a). The binding capacity of Nb23 and Nb23:EGFP was further assessed *in vitro* by dynamic light scattering (DLS). DLS analyses indicated that GFLV was monodisperse with a particle diameter of 32 ± 2 nm (mean ± SD) that increased to 36 ± 2 nm and 44 ± 2 nm upon binding of Nb23 or Nb23:EGFP at saturation, respectively (Figure 1c). Altogether, our results indicate that Nb23 binds specifically to GFLV particles and tags, as large as GFP, do not interfere with its binding capacity *in vitro*.

### Nb23 confers resistance to GFLV upon stable expression in *N. benthamiana*

**Figure 2:**
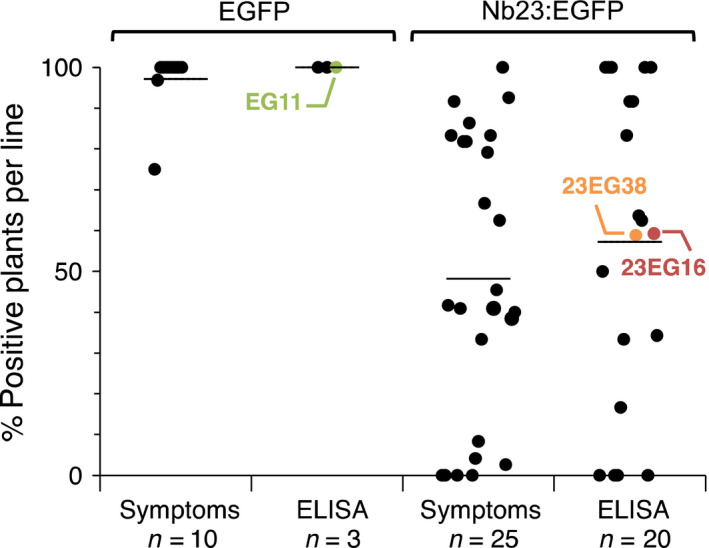
Evaluation of T1 transgenic *N. benthamiana* lines challenged with GFLV‐GHu. A total of 10 EGFP‐ and 25 Nb23:EGFP‐expressing lines, each consisting of 12–39 segregating plants, were mechanically inoculated with 300 ng of purified GFLV‐GHu, and infection was monitored by symptom assessment over 28 days and by GFLV DAS‐ELISA analysis at 28 dpi. Each dot on the graph corresponds to the percentage of infected plant per transgenic line. The numbers of transgenic lines analysed for symptoms and by DAS‐ELISA are indicated below each column (n). Horizontal bars indicate mean percentage values. Note that infection rates range from 0% to 100% for lines expressing Nb23:EGFP compared to 100% for lines expressing EGFP. The EGFP and Nb23:EGFP T1 lines selected to obtain T2 progeny are indicated.

**Figure 3:**
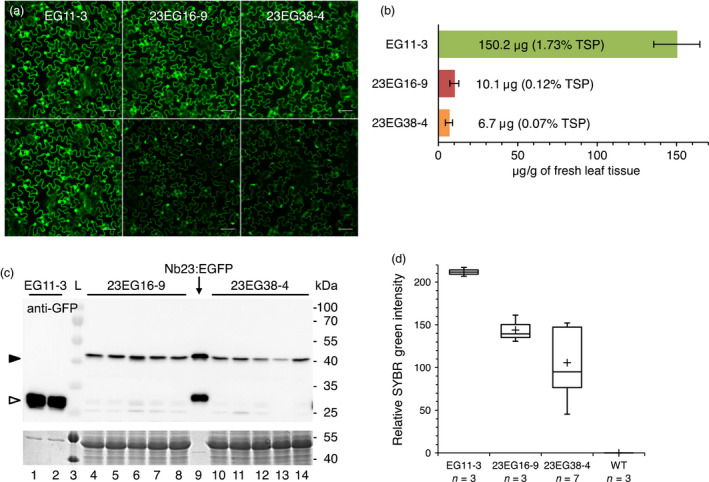
Characterization of homozygous T2 transgenic lines EG11‐3, 23EG16‐9 and 23EG38‐4. (a) Nb23:EGFP expressed from lines 23EG16‐9 (middle) and 23EG38‐4 (right) is found in the cytoplasm and nucleus of leaf epidermal cells, similarly to EGFP expressed from line EG11‐3 (left). Top row: equal fluorescence intensity. Bottom row: original images taken under identical acquisition settings. Note: fluorescence is highest in line EG11. Scale bars: 50 μm. (b) Quantification of Nb23:EGFP and EGFP in lines EG11‐3, 23EG16‐9 and 23EG38‐4. Fluorescence was quantified in soluble leaf extracts from transgenic lines and compared to fluorescence produced from known amounts of purified Nb23:EGFP and EGFP. TSP: total soluble proteins. (c) Immunoblot analysis of Nb23:EGFP and EGFP produced by lines EG11‐3, 23EG16‐9 and 23EG38‐4. TSP from leaves of EG11‐3 (0.25 mg fresh tissue equivalent, lanes 1, 2), 23EG16‐9 (7.5 mg fresh tissue equivalent, lanes 4 to 8) and 23EG38‐4 (7.5 mg fresh tissue equivalent, lanes 10 to 14) plants were probed with GFP antibodies. Semi‐purified Nb23:EGFP expressed from *E. coli* was used as size control (40 ng, lane 9) for Nb23:EGFP (black arrowhead) and EGFP (empty arrowhead). Bottom panel: equal loading control assessed by Coomassie blue staining of TSP. Lane 3: ladder with protein size indicated at the right. (d) Box plot representation of relative accumulation of EGFP/Nb23:EGFP transcripts determined by RT‐qPCR in lines EG11‐3, 23EG16‐9, 23EG38‐4 and WT. Plus signs correspond to mean values and whiskers to lowest or highest values within 1.5 interquartile range of the lower or higher quartile.

To assess the effect of Nb23:EGFP on GFLV *in vivo*, we produced transgenic *N. benthamiana* constitutively expressing Nb23:EGFP or EGFP only as a control. Initially, segregating T1 plants were challenged with GFLV isolate GHu because of its capacity to induce symptoms on *N. benthamiana* (Vigne *et al*., 2013). As expected, typical infection symptoms were observed in nearly 100% of the plants from the 10 independent lines constitutively expressing EGFP. The presence of GFLV in all plants from three of these lines, including line EG11, was confirmed by DAS‐ELISA (Figure 2). In contrast, infection rates among the Nb23:EGFP‐expressing lines were very variable whether determined on the basis of viral symptoms (25 lines scored) or by DAS‐ELISA (20 lines scored, Figure 2). By DAS‐ELISA, 11 lines were found to display intermediate levels of infection including lines 23EG16 and 23EG38 that showed approximately 60% infection rate, whereas five lines were fully infected and in the remaining four lines, GFLV was undetectable (Figure 2). These data on segregating T1 plants suggest that Nb23:EGFP expression confers resistance to GFLV. Nb23:EGFP‐expressing T1 lines 23EG16 and 23EG38, which displayed intermediate levels of resistance, as well as the susceptible control line EG11 expressing EGFP (Figure 2), were further selected for the production of homozygous T2 lines (EG11‐3, 23EG16‐9 and 23EG38‐4) due to their near 3:1 segregating ratio based on fluorescence phenotype. In the three selected lines, transgene expression in expanded leaves was analysed by confocal microscopy (Figure 3a), fluorimetry (Figure 3b), immunoblotting (Figure 3c) and RT‐qPCR (Figure 3d). Confocal microscopy revealed that both EGFP and Nb23:EGFP were homogenously expressed within leaves although to various levels as judged by differences in fluorescence intensity between the three lines (Figure 3a). Intracellularly, fluorescent proteins displayed a nuclear and cytoplasmic localization in leaf epidermal cells (Figure 3a). Fluorimetry measurements confirmed that average fluorescence in EG11‐3 plants was 14.9 and 22.4 times higher than in 23EG16‐9 and 23EG38‐4 plants, respectively (Figure 3b). Protein accumulation in lines EG11‐3, 23EG16‐9 and 23EG38‐4 was estimated to represent 1.73%, 0.12% and 0.07% of total soluble proteins (TSP), respectively (Figure 3b). Immunoblotting with anti‐GFP antibodies confirmed the clear difference in accumulation of Nb23:EGFP and EGFP between lines and further showed that full‐length proteins accumulated to similar levels within plants from a given line as expected for homozygous lines (Figure 3c). In contrast to protein accumulation determined by fluorimetry or immunoblotting, only a 1.5‐ to 2.0‐fold difference in mean relative accumulation of EGFP vs Nb23:EGFP transcripts was measured by RT‐qPCR (Figure 3d). This suggests that translational or post‐translational differences significantly contribute to the reduced recombinant protein accumulation levels in Nb23:EGFP‐ vs EGFP‐expressing plants. It is likely that the plant cytoplasm is less favourable for Nb stability than the naturally oxidizing environment found upon immunoglobulin secretion in mammals (Saerens *et al*., 2008).

**Figure 4:**
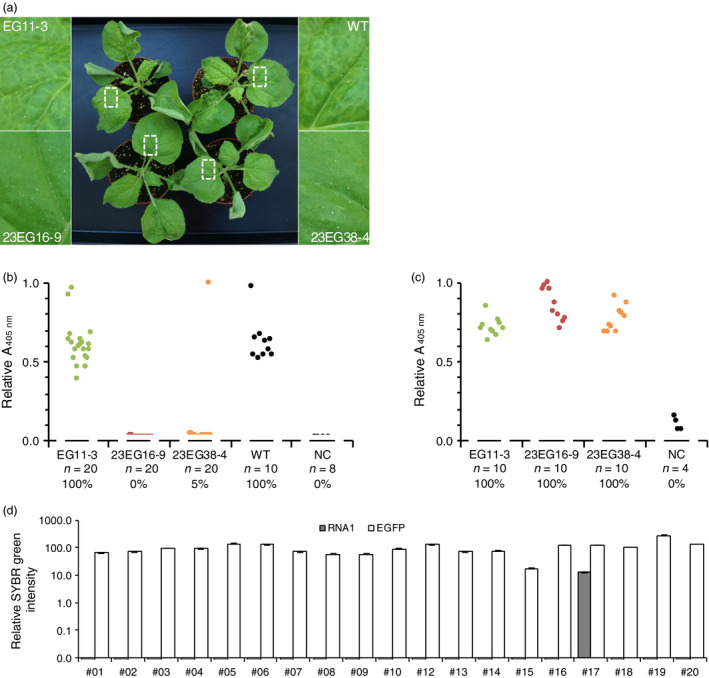
Evaluation of the resistance of transgenic *N. benthamiana* T2 lines 23EG16‐9 and 23EG38‐4 to infection by GFLV‐GHu and ArMV‐S. (a) Assessment of GFLV‐GHu symptoms of mechanically inoculated 23EG16‐9, 23EG38‐4, EG11‐3 and WT *N. benthamiana* at 7 dpi. WT and EG11‐3 lines showed mosaic symptoms on systemic leaves whereas 23EG16‐9 and 23EG38‐4 lines remained asymptomatic. (b) GFLV and c, ArMV DAS‐ELISA performed at 21 dpi on upper uninoculated leaves from EG11‐3, 23EG16‐9, 23EG38‐4 and WT plants. Each dot corresponds to a single plant sample and represents the mean relative absorbance at 405 nm of experimental duplicates. Number of plants tested (*n*) and percentage of infection (%) are provided below each column. Noninoculated plants were used as negative control (NC). d, Relative accumulation of GFLV RNA1 transcripts (grey bars) and EGFP/Nb23:EGFP transcripts (white bars) determined by RT‐qPCR in individual plants from line 23EG38‐4. Error bars show standard deviation of experimental triplicates. RNA1 is detected in plant #17 only. All inoculations were performed with 300 ng of purified viruses.

**Figure 5:**
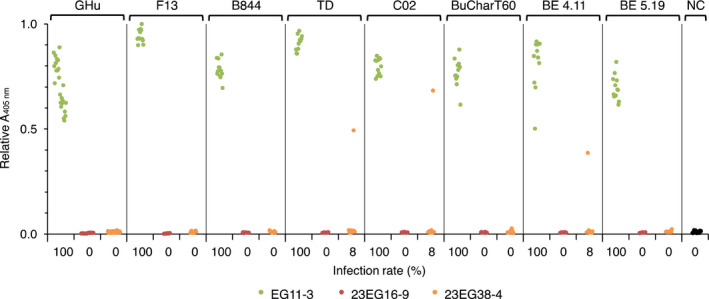
Evaluation of the resistance of T2 lines 23EG16‐9 and 23EG38‐4 to infection by various GFLV isolates. EG11‐3 (green), 23EG16‐9 (red) and 23EG38‐4 (orange) plants were mechanically inoculated with saps from *C. quinoa* plants infected with GFLV isolates GFLV‐GHu, GFLV‐F13, GFLV‐B844, GFLV‐TD, GFLV‐CO2, GFLV‐BUCharT60, GFLV‐BE4.11 and GFLV‐BE5.19 and apical noninoculated leaves analysed by DAS‐ELISA at 21 dpi. Each dot corresponds to a single plant sample and represents the mean relative absorbance at 405 nm of experimental duplicates. Noninoculated plants were used as negative control (NC). For each set of inoculation, a total of 12 plants were tested, except for GFLV‐GHu (24 plants) and NC (17 plants). Percentage of infections is indicated below each column. Note that line 23EG16‐9 is 100% resistant to all GFLV isolates. Altogether, only three plants of 108 tested positive for GFLV in line 23EG38‐4.

**Figure 6:**
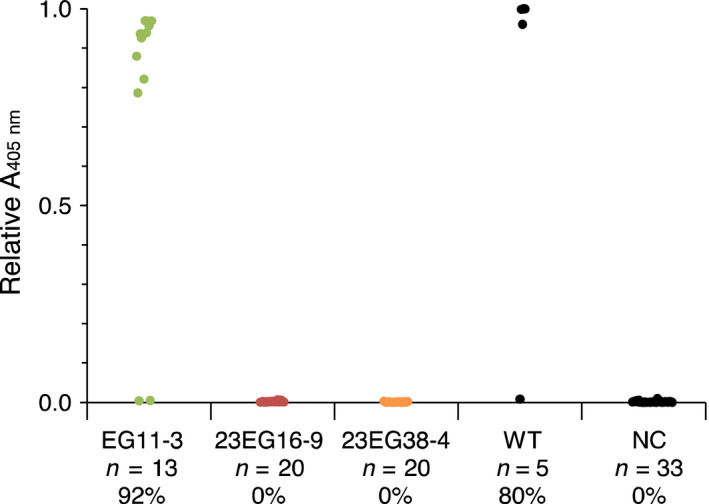
Evaluation of the resistance of T2 lines 23EG16‐9 and 23EG38‐4 to GFLV transmission by *X. index*. EG11‐3 (green), 23EG16‐9 (red), 23EG38‐4 (orange) and WT plants were grown in contact with viruliferous nematodes harbouring GFLV‐F13. Plants of each genotype maintained in contact with aviruliferous *X. index* were included as negative control (NC). GFLV DAS‐ELISA was performed on leaves 16 weeks after initial contact with nematodes. Relative absorbance values are indicated for each plant per lines. Each dot corresponds to a single plant and represents the mean relative absorbance at 405 nm of experimental duplicates. Number of plants tested (*n*) and percentage of infection (%) are provided below each column. Note the absence of infection in lines 23EG16‐9 and 23EG38‐4.

To evaluate the susceptibility of homozygous T2 lines to infection, plants were challenged with GFLV and ArMV isolates, either by mechanical inoculation or upon transmission of GFLV by *X. index,* the specific vector of GFLV. When challenged with 300 ng of GFLV‐GHu, all EG11‐3 plants showed symptoms similar to those seen in wild‐type plants (Figure 4a) and tested GFLV positive by DAS‐ELISA (Figure 4b), whereas only one plant of 20 in line 23EG38‐4 and none of 20 in line 23EG16‐9 was infected at 21 days postinoculation (dpi) (Figure 4b). RT‐qPCR analysis confirmed the single infection event in line 23EG38‐4 and further revealed that infection was not due to partial or complete loss of Nb23:EGFP expression as relative accumulation of RNA encoding Nb23:EGFP was similar in all plants (Figure 4d). In accordance with the inability of Nb23 to recognize ArMV (Figure 1b), all plant lines challenged with ArMV‐S tested positive by DAS‐ELISA against ArMV (Figure 4c). In addition, whether challenged with eight different GFLV isolates (Figure 5), GFLV viral RNA (Figure S3) or with viruliferous nematodes (Figure 6), control line EG11‐3 showed high susceptibility, contrarily to lines 23EG16‐9 and 23EG38‐4 that displayed resistance to GFLV. When combined, a total of 158 plants from each 23EG16‐9 and 23EG38‐4 lines were challenged with GFLV under various inoculation conditions (Table 1). Remarkably, while GFLV was detected at only low frequency in line 23EG38‐4 (3.2%, five of 158 plants, Table 1), all plants from line 23EG16‐9 remained virus free and thus were fully resistant to infection (0.0%, 0 of 158 plants, Table 1). It is therefore concluded that constitutive expression of Nb23:EGFP confers broad‐range resistance to GFLV but not to ArMV in transgenic *N. benthamiana*. Most remarkably, this resistance is effective whatever inoculation method is used including upon transmission of GFLV by its natural vector *X. index*. To our knowledge, this is the first evidence of strong resistance to GFLV transmission by nematodes.

**Table 1:** Nb23:EGFP confers broad‐range resistance to GFLV but not to ArMV in transgenic *N. benthamiana*

Isolate	Inoculation	EG11‐3			23EG16‐9			23EG38‐4		
		Number of plants tested	Number of plants infected	Infection rate	Number of plants tested	Number of plants infected	Infection rate	Number of plants tested	Number of plants infected	Infection rate
GFLV‐GHu	300 ng	20	20	100%	20	0	0%	20	1	5%
	Sap	24	24	100%	24	0	0%	24	0	0%
	RNA 360 ng	10	10	100%	10	0	0%	10	1	10%
GFLV‐F13	Sap	12	12	100%	12	0	0%	12	0	0%
	Nematodes	13	12	92%	20	0	0%	20	0	0%
GFLV‐B844	Sap	12	12	100%	12	0	0%	12	0	0%
GFLV‐TD	Sap	12	12	100%	12	0	0%	12	1	8%
GFLV‐CO2	Sap	12	12	100%	12	0	0%	12	1	8%
GFLV‐BUCharT60	Sap	12	12	100%	12	0	0%	12	0	0%
GFLV‐BE 4.11	Sap	12	12	100%	12	0	0%	12	1	8%
GFLV‐BE 5.19	Sap	12	12	100%	12	0	0%	12	0	0%
**Total GFLV**		**151**	**150**	**99.3%**	**158**	**0**	**0.0%**	**158**	**5**	**3.2%**
ArMV‐S	300 ng	10	10	100%	10	10	100%	10	10	100%

T2 transgenic lines EG11‐3, 23EG16‐9 and 23EG38‐4 were mechanically inoculated as indicated and upper uninoculated leaves were tested for GFLV or ArMV presence by DAS‐ELISA at 21 dpi, except for nematode inoculation for which roots were left in contact with viruliferous nematodes for 6 weeks and DAS‐ELISA performed on leaves 16 weeks postcontact.

### Virus neutralization occurs early during infection before cell‐to‐cell movement

**Figure 7:**
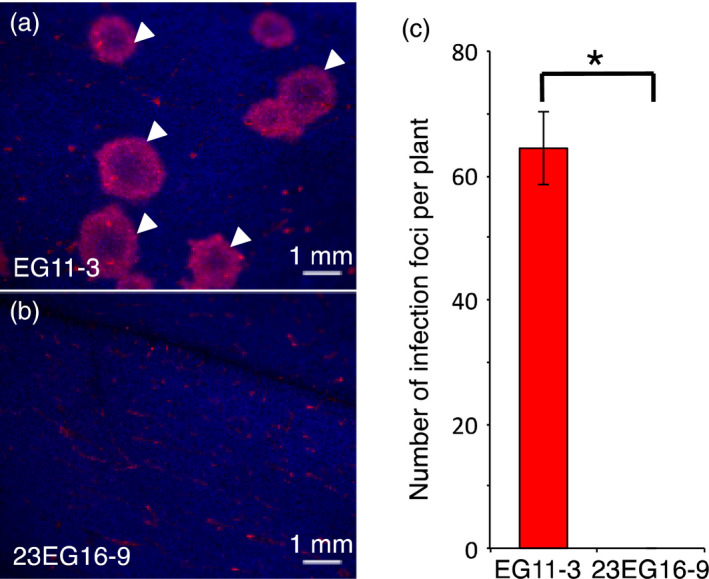
Virus neutralization occurs early during infection before cell‐to‐cell movement. Fluorescence images from (a) EGFP‐ and b, Nb23:EGFP‐expressing *N. benthamiana* leaves inoculated with a recombinant GFLV encoding TagRFP, at 6 dpi. Arrowheads point at typical GFLV infection foci that appear as doughnut‐shaped red structures. Blue colour corresponds to chlorophyll and faint red background in (a and b) to autofluorescence generated by physical damage upon mechanical inoculation. (c) Average number of infection foci calculated from three different leaves and three independent plants from lines EG11‐3 and 23EG16‐9. Values are means ± SD (*n *=* *9). The asterisk indicates statistically significant differences (Student *t*‐test, *P *=* *0.002).

The absence of GFLV in the upper uninoculated leaves suggests that virus neutralization occurs early during infection, before systemic movement is initiated. To address more precisely the stage at which GFLV infection is arrested, we monitored infection using a recombinant GFLV encoding TagRFP (Schmitt‐Keichinger *et al*., 2017). Whereas numerous infection foci (64.3 ± 5.9 foci) were counted on three inoculated leaves from three independent EG11‐3 plants (Figure 7a and c), no evidence of infection in equivalent leaves from 23EG16‐9 plants was detected (Figure 7b and c). Monitoring the same inoculated leaves by fluorescence microscopy at higher magnification did not allow the detection of red fluorescent cells as would have been the case upon confinement of GFLV to individual inoculated cells (not shown). Altogether, our results indicate that Nb23‐mediated GFLV antiviral activity is potent and virus neutralization occurs at early stages of infection before cell‐to‐cell movement is initiated.

### Nb23‐derived resistance to GFLV observed in *N. benthamiana* also applies to grapevine

**Figure 8:**
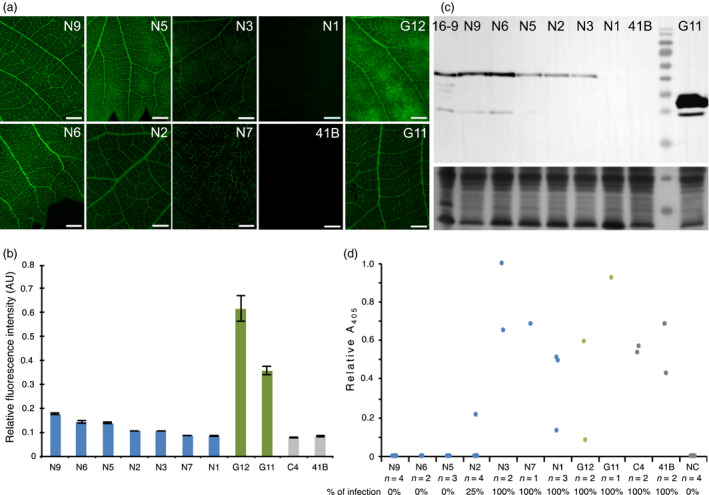
Nb23:EGFP confers resistance to GFLV in transgenic grapevine. (a) Fluorescence images from Nb23:EGFP‐expressing (N9, N6, N5, N2, N3, N7 and N1), EGFP‐expressing (G11, G12) and control (41B) grapevine leaves. Images were taken under identical exposure conditions. bars = 2 mm. (b) Relative quantification of fluorescence of leaves presented in panel a and from line C4. Note that fluorescence measured in Nb23:EGFP‐expressing lines N9, N6 and N5 is highest but much lower than in EGFP‐expressing lines G11 and G12. (c) Immunoblot analysis of leaf protein extracts from transformed grapevine lines N9, N6, N5, N3, N2, N1 and G11 with GFP antibodies. Untransformed control grapevine leaves (lane 41B) and *N. benthamiana* leaves from line 23EG16‐9 (lane 16‐9) were used as negative and positive control, respectively. Bottom panel: equal loading was assessed by Coomassie blue staining of total proteins (40 μg of protein per lane) d, GFLV DAS‐ELISA performed on micrografts at 45 days postacclimatization. Number of micrografts tested (*n*) and percentage of infections are indicated for each line. Note that all micrografts from lines N9, N6 and N5 are ELISA negative. NC: negative control (uninfected plants).

Whether the antiviral activity observed in the model plant *N. benthamiana* also applies to grapevine, the natural host of GFLV, was addressed in transgenic rootstocks of the 41B genotype. Regeneration from embryogenic calli resulted in the production of seven independent transformed lines encoding Nb23:EGFP named N1, N2, N3, N5, N6, N7 and N9; hereafter, two transformed lines encoding EGFP (G11 and G12) and a single untransformed line (C4) were used as a control. Epifluorescence imaging of leaves from transformed grapevines revealed differences in EGFP fluorescence intensity with decreasing Nb23:EGFP expression levels detected from lines N9 (highest expressing line), N6, N5, N2, N3, N7 to line N1 in which barely any fluorescence was detected (Figure 8a, b). Fluorescence appeared highest in the vasculature but was also detected all over the leaf surface in all lines except in line N7 in which the fluorescence seemed restricted to the vascular tissues (Figure 8a). As with *N. benthamiana*, EGFP was expressed to much higher levels than Nb23:EGFP in corresponding lines whether protein accumulation was measured by fluorescence quantification (Figure 8b) or by immunoblotting using GFP antibodies (Figure 8c). The latter analysis also confirmed that Nb23:EGFP was expressed as a full‐length protein, and accumulation levels in lines N9 and N6 was found similar to those detected in the *N. benthamiana* line 23EG16‐9 (Figure 8c).

Virus mechanical inoculation on grapevine being inefficient (Valat *et al*., 2003b), resistance was assessed upon micrografting of transgenic canes onto GFLV‐infected grapevine material. All canes expressing EGFP (*n* = 3), regenerated control canes C4 (*n* = 2) and canes from untransformed control lines 41B (*n* = 2) as well as canes from lines N3 (*n* = 2), N7 (*n* = 1) and N1 (*n* = 3) expressing lower levels of Nb23:EGFP tested GFLV positive by DAS‐ELISA, 6 weeks postgrafting (Figure 8d). In contrast, GFLV remained undetectable in all canes from lines expressing highest levels of Nb23:EGFP N9 (*n* = 4), N6 (*n* = 2) and N5 (*n* = 3) (Figure 8d), demonstrating that Nb23‐mediated resistance to GFLV observed in *N. benthamiana* also applies to grapevine and positively correlates with Nb23:EGFP accumulation levels.

## Discussion

Because of their outstanding and often unique properties, including small size approximately 15 kDa, hence a tenth the size of a conventional immunoglobulin, high solubility and stability as well as capacity to bind epitopes inaccessible to conventional antibodies, Nbs derived from camelid heavy‐chain‐only antibodies have proven to be of tremendous biotechnological interest (Hamers‐Casterman *et al*., 1993; Muyldermans, 2013). First discovered in 1993 (Hamers‐Casterman *et al*., 1993), their use covers a wide range of applications such as bioimaging, prophylactic or therapeutic vaccines, diagnosis for various human and animal diseases such as cancer, Alzheimer or viral diseases (De Meyer *et al*., 2014; Wang *et al*., 2016). In contrast, their use in plant biology is rather limited (Wang *et al*., 2016). It is only recently that Ghannam *et al*. (2015) suggested their potential as antiviral molecules against plant viruses by showing that Nbs directed against BBMV attenuated viral spreading upon transient expression in *Vicia faba* leaves. Here, we confirm this hypothesis by showing that a single Nb with specificity to GFLV is able to confer strong resistance to the cognate virus when stably expressed in transgenic plants. This conclusion applies not only to the model plant *N. benthamiana* but also to the economically relevant crop grapevine for which GFLV is a very serious pest. To the best of our knowledge, it is the first report of Nb‐mediated resistance in transgenic plants and perhaps more importantly, the first compelling evidence of resistance to GFLV in *Vitis* (Basso *et al*., 2017).

The use of antibodies to confer resistance against plant pathogens has been reported previously (for review see Safarnejad *et al*. (2011) including for GFLV and ArMV in *N. benthamiana* (Nölke *et al*., 2009). In general, best results were obtained with single‐chain variable fragments (scFvs) rather than full‐length immunoglobulins (Schillberg *et al*., 1999). Here, we show that Nb23 is functional and accumulates to concentrations of 0.12% (line 23EG16‐9) and 0.07% (line 23EG38‐4) of TSP in the cytoplasm of resistant *N. benthamiana* cells and to similar levels in resistant grapevine lines. Similar expression levels (up to 0.1% of TSP) were achieved *in N. benthamiana* expressing scFvGFLVcp‐55 directed against GFLV (Nölke *et al*., 2009), suggesting that Nb23 is as potent as scFvGFLVcp‐55 to confer resistance. Considering our results with GFLV and those of Ghannam *et al*. (2015) with BBMV, it is tempting to speculate that antiviral activity is a common property of Nbs directed against plant viruses and they should therefore be considered as a reliable source of resistance. In addition, considering the difficulties often encountered to generate functional scFvs (Fiedler *et al*., 1997; Safarnejad *et al*., 2011), which are circumvented with Nbs due to their intrinsic single‐domain origin and structural specificities (Muyldermans, 2013), Nbs should be considered superior to other antibodies‐derived products when aiming at generating virus‐resistant plants.

Here, we describe the production of 23 different Nbs directed against GFLV belonging to 11 different families and thus potentially able to recognize up to 11 different epitopes (Muyldermans, 2013). The choice of Nb23 (family 1, Figure S1) was essentially driven by its capacity to recognize numerous GFLV isolates *in vitro* (Figure 1) and hence for its potential to confer broad‐range resistance to GFLV, which we demonstrated. Whether antiviral activity is equivalent for all GFLV‐specific Nbs is unknown at this stage. However, considering Nbs from family 1 are likely recognizing the same epitope suggests that they all possess similar antiviral activity. In contrast to Nölke *et al*. (2009) in which scFvGFLVcp‐55 was shown to cross‐react with ArMV and to confer enhanced tolerance to ArMV, no cross‐reactivity was detected for Nb23 with ArMV and consequently transgenic lines expressing Nb23 displayed full susceptibility to ArMV.

In this work, we also describe the production of a total of 25 lines expressing Nb23:EGFP (Figure 2) of which only lines 23EG16‐9 and 23EG38‐4 were characterized up to T2 stage contrarily to the other lines that presented complex segregation profiles probably as a consequence of multiples insertions of the transgene. It is remarkable to notice that the vast majority of lines were resistant to GFLV, some up to 100% already at the T1 generation. Considering transgenic plants were selected on the basis of their fluorescence and therefore Nb23:EGFP expression, we assume that resistance is directly linked to the expression level of the transgene. However, contrarily to most other studies with scFvs (Boonrod *et al*., 2004; Nölke *et al*., 2009; Schillberg *et al*., 2000; Villani *et al*., 2005), we were unable to correlate the degree of virus resistance (i.e. virus load or delay in symptom appearance) to Nb23 accumulation levels, plant being either symptomatic and ELISA positive or asymptomatic and ELISA negative. Best example is provided by line 23EG38‐4 that showed high degree although not complete resistance to GFLV (3.2% infection rate, 158 plants challenged, Table 1). It is likely that the five plants of line 23EG38‐4 in which GFLV was detected despite appropriate expression of the transgene (Figure 4d) correspond to cases where the virus succeeded in overcoming resistance as a consequence of a mutation affecting the Nb23 epitope. Structural studies aiming at precisely identifying the Nb23 epitope and sequencing of the CP from putative GFLV escape variants for the presence of mutations explaining the loss of resistance shall be addressed elsewhere.

Finally, our results raise also the question of the resistance mechanism. As resistant transgenic plants showed no evidence of systemic spread of the virus, neither the occurrence of infection foci nor the presence of single infected cells on inoculated leaves indicates that Nb23 blocks GFLV very early during the infection process, before cell‐to‐cell movement of the virus. Virus neutralization may be the consequence of a stabilizing effect exerted by Nb23 upon binding to the capsid, preventing its disassembly and thereby the release of the viral RNA and consequently the initiation of the replication cycle. Nbs with stabilizing activity have been reported for poliovirus, but strict correlation between virus neutralization and stabilizing activity could not be established (Schotte *et al*., 2014). Another possibility may consist in inhibition of capsid formation due to early CP tethering prior to virion assembly. Considering that GFLV moves from cell to cell as entire virions via tubules that form within plasmodesmata (Laporte *et al*., 2003; Ritzenthaler *et al*., 1995), the interference of Nb23 with capsid assembly would ineluctably prevent the spread of the virus to neighbouring cells. Finally, one cannot rule out the possibility that Nb23 may interfere with CP folding resulting in the cytosolic accumulation of aggregated or misfolded proteins that could act as a signal for CP targeting to degradation pathways (Liu and Bassham, 2012) and thereby virus clearance. Of course, these hypotheses should not be considered as exhaustive or mutually exclusive and further studies are needed.

In conclusion, we have shown that Nb‐mediated resistance can be used to confer resistance to GFLV in *N. benthamiana* and more importantly in grapevine where there is an urgent need to find sustainable solutions to the increasing problem of fanleaf degeneration in cultivated vineyards. The proof of concept being established, our work shall pave the way for the creation of novel virus‐resistant varieties of agriculturally important crops. Whether it will help to improve the public acceptance of genetically modified crops is a long debate that extends far beyond science.

## Experimental procedures

### Virus isolates

GFLV and ArMV isolates used here are GFLV‐GHu (Vigne *et al*., 2013), GFLV‐F13 (Pinck *et al*., 1988), GFLV‐B844 (Legin *et al*., 1993), GFLV‐TD (Schellenberger *et al*., 2011b), GFLV‐CO2 (Vigne *et al*., 2004a), GFLV‐BuCharT60, GFLV‐BE 4.11, GFLV‐BE 5.19 and ArMV‐S (Loudes *et al*., 1995).

### Immunization, Nbs library construction and screening

GFLV‐specific Nbs were generated according to Muyldermans *et al*. (2009). Briefly, a camel (*Camelus dromedarius*) was immunized six times subcutaneously at weekly intervals with 100 μg of purified GFLV‐F13 according to standard immunization protocols. After immunization, total RNA was extracted from isolated peripheral blood lymphocytes and mRNAs reverse‐transcribed to cDNA. The regions encoding variable fragments of heavy‐chain‐only antibodies were amplified in two subsequent PCR, cloned into the pHEN4 phagemid vector and transformed into *E. coli* TG1. The resulting Nbs library was screened by phage display for GFLV‐specific binders in three consecutive biopanning rounds against 10 μg of purified GFLV‐F13 each. Sequences of GFLV‐specific Nbs were obtained following the isolation of individual clones from the enriched library by a phage ELISA approach performed against 100 ng of purified GFLV‐F13.

### Expression and purification of Nbs from *E. coli*

GFLV‐specific Nbs coding sequences were subcloned into the pHEN6 (Conrath *et al*., 2001) expression vector as a BstEII/PstI fragment adding a N‐terminal PelB signal peptide sequence for periplasmic targeting and a C‐terminal 6‐His‐tag for purification. Production of 6‐His‐tagged Nbs constructs was performed by expression in *E. coli* WK6 grown in Terrific Broth medium and induced overnight with 1 mm IPTG at 28 °C.

An additional C‐terminal Strep‐tag II (Trp‐Ser‐His‐Pro‐Gln‐Phe‐Glu‐Lys) was added for Nbs used in ELISA. To do so, Nbs coding sequences were amplified by PCR and amplicons were introduced by Gateway cloning into the pDONR/Zeo vector (Thermo Fisher Scientific, France) which was further recombined into the p0GWA expression vector (Busso *et al*., 2005). Large‐scale production of Strep II‐tagged Nbs constructs was performed by expression in *E. coli* BL21 (DE3) grown overnight at 23 °C in auto‐inducing ZYP50502 medium.

Nbs were extracted from periplasm by osmotic shock (Habib *et al*., 2013) and purified at 4 °C by immobilized metal ion chromatography (IMAC) on a 1 mL Protino Ni‐NTA column (Macherey‐Nagel, France) using 500 mm imidazole in running buffer (50 mm Tris, 300 mm NaCl, 5% glycerol, pH 8.0) for elution, followed by size exclusion chromatography (SEC) on a Hiload 16/60 Superdex75 prep grade column (GE Healthcare Life Science, France) in 1X phosphate buffer saline. Purity of eluted proteins was assessed by Coomassie blue staining of denatured Nbs in denaturing Tris–tricine polyacrylamide gel. Purification yields were estimated from absorbance at 280 nm based on extinction coefficients computed from Nbs composition.

### DAS‐ELISA assessment of Nb23 reactivity against GFLV strains

Plants at the four‐ to six‐leaf stage were mechanically inoculated with crude saps from *Chenopodium quinoa* infected with various GFLV isolates. Apical noninoculated leaves were ground in extraction buffer (35 mm Na_2_HPO_4_, 15 mm KH_2_PO_4_, pH 7.0) in a 1:5 w/v ratio. Virus detection was performed on clarified extracts by DAS‐ELISA using anti‐GFLV or anti‐ArMV polyclonals (Bioreba AG, Switzerland) diluted 1000‐fold in coating buffer (15 mm Na_2_CO_3_, 35 mm NaHCO_3_, pH 9.6) as capture antibody and the Strep II‐tagged Nb23 at 1 μg/mL in conjugate buffer (10 mm PBS, 0.1% w/v bovine serum albumin (BSA), 0.05% v/v Tween‐20, pH 7.4) as detection antibody. For development, streptavidin–alkaline phosphatase (Jackson Immunoresearch, Suffolk, UK) at 50 ng/mL in conjugate buffer was used in conjunction with para‐nitrophenyl phosphate (Interchim, France) at 1 mg/mL in substrate buffer (1 m diethanolamine, pH 9.8). Negative control consisted of noninoculated healthy plants. Absorbance at 405 nm (A_405 nm_) was recorded after one hour, and samples with mean A_405 nm_ values exceeding by a factor of 2.4 those of negative controls were considered positive. Results are presented as mean absorbance of experimental duplicates ± standard error normalized against maximum assay value.

### *N. benthamiana* transformation and production

Nb23 was cloned in frame to the N‐terminus of EGFP with a Gly3SerGly3 linker sequence into the pEAQ‐HT‐DEST3 plant expression vector (Sainsbury *et al*., 2009). EGFP was first introduced into the pHEN6‐Nb23 vector as an EcoRI/BstEII fragment following a PCR amplification. The Nb23:EGFP gene was then reamplified by PCR and introduced by Gateway cloning into the pDONR/Zeo vector, which was further recombined into the pEAQ‐HT‐DEST3 vector. A control consisting of pEAQ‐HT‐DEST3‐EGFP was included.

The resulting vectors were transferred into *Agrobacterium tumefaciens* GV3101::pMP90 and used at A_595 nm_ = 0.1 for agrotransformation of *N. benthamiana* leaves. After 3 days, expression of fluorescence was checked for EGFP expression with an Axio Zoom V16 macroscope (Zeiss, Germany) and sterilized infiltrated leaf segments were placed in a growth chamber (16 h light/8 h dark, 25 °C) onto Murashige and Skoog medium ((MS, Duchefa, The Netherlands), 10 mm NH_4_NO_3_, 1× MS vitamin solution (Sigma‐Aldrich, France), 3% w/v sucrose, 0.05 μg/mL 1‐naphthalene acetic acid, 2 μg/mL 6‐benzyl‐aminopurine, 0.8% w/v agar, 150 μg/mL kanamycin, 500 μg/mL carbenicillin, pH 5.8). Calli were subcultured every week onto fresh medium and shoots excised 3–4 weeks later transferred onto rooting medium (1/2 MS medium, 1.5% w/v sucrose, 0.5× MS vitamin solution, 0.8% w/v agar, 150 μg/mL kanamycin, 500 μg/mL carbenicillin, pH 5.8) until plantlets could be acclimatized and established in soil. Regenerated T0 plants were self‐pollinated through T2 generation.

### Nb23:EGFP purification

For Nb23:EGFP production, the whole Nb23:EGFP:6‐His coding sequence was cloned into the pET‐22b(+) expression vector (Novagen, Madison) as a NdeI/XhoI fragment subsequent to a PCR amplification using pEAQ‐HT‐DEST3‐Nb23:EGFP as template. Expression was performed in *E. coli* SHuffle T7 Express (New England Biolabs, France) grown in TB medium and induced overnight with 0.1 mm IPTG at 20 °C. Pelleted cells resuspended in PB‐NaCl buffer (10 mm phosphate buffer, 300 mm NaCl, pH 7.4) were lysed by sonication (80% amplitude for 2 min with 13 mm diameter probe, Vibra‐Cell VCX 500 (Sonics & Materials Inc) before purification of cytoplasmic extract. The purity of the eluted proteins was assessed by Coomassie blue staining after denaturing Tris–tricine polyacrylamide gel electrophoresis.

### Quantification of recombinant proteins in *N. benthamiana* by fluorimetry

Three youngest apical leaves of 6–7 weeks old *N. benthamiana* were homogenized in extraction buffer (200 mm Tris‐HCl, 300 mm NaCl, 100 mm ascorbic acid, 2.5% w/v polyvinylpolypyrrolidone, complete protease inhibitor cocktail (Roche, France), pH 7.0) at a 1:2 w/v ratio. Cell debris were removed by centrifugation at 20 000 *g* for 20 min at 4 °C, and TSP concentrations were determined using the Bio‐Rad protein assay following manufacturer's instructions with BSA as standard.

Fluorescence intensity was recorded in a FLUOstar Omega plate reader (BMG Labtech, Germany) equipped with 485 ± 12 nm excitation and 520 ± 25 nm emission filters, on 100 μL of soluble extracts in a white flat‐bottom polystyrene plate (Greiner Bio One, Austria). Wild‐type *N. benthamiana* extracts were used as blank and known amount of purified Nb23:EGFP for fluorescent titration.

### Fluorescence quantification of recombinant proteins in grapevine leaves

Two leaves from each line were imaged under identical conditions using a Axio Zoom V16 stereomicroscope (Zeiss, Germany) equipped with appropriate excitation and emission filters for the visualization of EGFP and chlorophyll. The fluorescence was quantified using ImageJ (rsb.info.nih.gov/ij/) on six randomly chosen areas of identical surface (8176636 pixels) for each leaf.

### Immunoblotting

For *N. benthamiana,* acetone‐precipitated TSP were used. For grapevine, proteins were extracted according to Hurkman and Tanaka (1986). Proteins were heated in denaturing buffer, separated by Tris‐glycine SDS‐PAGE and transferred onto a polyvinylidene difluoride membrane using the TransBlot Turbo transfer system (Bio‐Rad, France). After incubation in blocking buffer (10 mm PBS, 0,1% v/v Tween‐20, 5% w/v skim milk), proteins were sequentially probed with rabbit anti‐GFP IgGs (Sigma‐Aldrich, France) at 0.1 μg/mL in blocking buffer and goat anti‐rabbit IgGs conjugated to horseradish peroxidase (Thermo Fisher Scientific, France) at 0.1 μg/mL in blocking buffer. Immunolabelled proteins were detected by enhanced chemiluminescence (ECL) using the Lumi‐Light^PLUS^ kit (Roche, France) and the Fusion FX imaging system (Vilber Lourmat GmbH, Germany).

### Dynamic Light Scattering (DLS)

Viral particles were purified as described previously (Schellenberger *et al*., 2011a). Mean particle diameters and polydispersity of GFLV‐F13 alone or complexed to Nb23 or to Nb23:EGFP was estimated by DLS using a Zetasizer NanoZS (Malvern, France) and Nanostar (Wyatt, CA). Five successive measurements were performed using three independent virus and protein preparations with virus at 0.1 mg/mL in Tris buffer (50 mm Tris, 100 mm NaCl, pH 8.3), Nb23 at 0.1 mg/mL and Nb23:EGFP at 0.9 mg/mL. Scattered intensities were recorded at 20 °C and data were processed with DTS software (www.dtssoftware.com, version 6.01) or DYMAMICS (www.wyatt.com/products/software/dynamics.html, version 7.1.8.93), respectively. All particles were monodisperse.

### Reverse Transcriptase‐qPCR (RT‐qPCR) analyses

GFLV RNA1 and EGFP/Nb23:EGFP transcripts were quantified by RT‐qPCR relatively to the expression of cyclin‐dependent kinase homolog (GI:849067, *N. tabacum*), elongation factor 1 alpha (GI:37783254) and actin (GI:380505031) genes from *N. benthamiana* used as internal controls due to their stability assessed by GeneNorm (Vandesompele *et al*., 2002) and NormFinder (Andersen *et al*., 2004) algorithms. Total RNA was isolated at 21 dpi from approximately 17 mg of noninoculated apical leaves ground at a 1 : 30 w/v ratio in TLES buffer (100 mm Tris, 100 mm LiCl, 10 mm EDTA, 0.1% w/v SDS, pH 8.0) followed by a water‐saturated phenol and phenol chloroform extraction before precipitation with 2 m LiCl. cDNA was generated according to manufacturers' instructions from 1 μg of DNaseI‐treated total RNA using 2.5 μm Oligo(dT)18 primer (Thermo Fisher Scientific, France) and SuperScript III Reverse Transcriptase (Thermo Fisher Scientific, France). PCR was performed in triplicates using 0.5 μL of reverse transcription reaction and 2.5 μm gene specific primers in a total volume of 10 μL LightCycler 480 SYBR Green Master I mix on a LightCycler 480 system (Roche, France) with cycling conditions of 5‐min denaturation at 95 °C followed by 40 cycles at 95 °C for 10 s, 60 °C for 15 s and 72 °C for 15 s. Relative gene expression levels were calculated by means of the linear regression of efficiency method using LinRegPCR software (version 2013.0) (Ruijter *et al*., 2009). Primer sequences are available upon request.

### Fluorescence microscopy

For cellular‐level scale observations, water‐mounted leaf discs of six‐ to 7‐week‐old *N. benthamiana* plants were imaged with a LSM 780 laser scanning confocal microscope attached to an observer Z1 microscope (Zeiss, Germany) equipped with a 20×/0.8 Plan‐Apochromat objective and using excitation and emission wavelength filters set to 488 nm and 499–521 nm.

For macroscopic observations, leaves were imaged with a MacroFluo Z16 APO(A) macroscope (Leica, Germany) using excitation and emission wavelength filters of 538–562 nm and 570–640 nm for red channel. Images were processed using Zen 2011 imaging software (Zeiss) and Photoshop CS5 (Adobe).

### Inoculations

Plants were inoculated either mechanically or via nematodes. Mechanical inoculations were performed with purified virus, with saps of infected *C. quinoa* or with purified viral RNA.

Nematode inoculation was performed by growing plants for 6 weeks in soil (3 : 1 : 1 v/v ratio of sand, loess and clay pebbles) containing ca. 300 viruliferous *X. index* per plant. GFLV ELISAs were performed at 16 weeks postcontact with nematodes on apical noninoculated leaves.

### Grapevine transformation

A friable embryogenic callus originated from the 41B rootstock cultivar was used for genetic transformation experiments as described by Romon *et al*. (2013) using the same pEAQ constructs than used for *N. benthamiana* transformation. After a 9‐ to 12‐month selection process, seven transgenic lines harbouring the Nb23:EGFP construct (lines N1, N2, N3, N5, N6, N7 and N9) and two transgenic lines harbouring solely the EGFP construct (lines G11 and G12) were isolated on the basis of their resistance to 50 μg/mL kanamycin. A wild‐type 41B rootstock and a regenerated plant named C4 issued from the same embryogenic callus in the absence of kanamycin were used as controls. All the lines were maintained *in vitro* as cuttings on McCown woody plant medium (McCown and Lloyd, 1981).

### *In vitro* micrografting

One‐node cuttings of nine transgenic grapevine lines and two control lines were *in vitro*‐grafted as scion onto GFLV‐F13‐infected Kober 5BB rootstock cultivar by cleft grafting under sterile conditions as described by Valat *et al*. (2003a). After 3 months of *in vitro* culture, rooted grafts were acclimatized in a growth chamber. The presence of GFLV in scions was assessed by DAS‐ELISA 45 days after acclimatization.

### Statistical analyses

For infection foci measurement, statistical analysis was performed by unequal variances two‐tailed unpaired Student's *t*‐test (*P* value = 0.002) and Shapiro–Wilk test for normality (*W *=* *0.87. *P* value = 0.12).

## Supporting information


**Figure S1** Phylogenetic tree of Nanobodies (Nbs) directed against GFLV.
**Figure S2** Unrooted phylogenetic tree reconstructed from the amino acid sequence of the CP protein of the eight GFLV isolates and ArMV‐S.
**Figure S3** Evaluation of the resistance of T2 lines 23EG16 9 and 23EG38 4 to infection by viral RNA.
