# Supplementary material for: Nanobody‐mediated resistance to Grapevine fanleaf virus in plants
Source: Plant Biotechnol J. 2017 Oct 6;16(2):660–71. doi: 10.1111/pbi.12819 (PMC5787842; doi:10.1111/pbi.12819)
Supplement: Supplementary file 1 — Figure S1 Phylogenetic tree of Nanobodies (Nbs) directed against GFLV. Figure S2 Unrooted phylogenetic tree reconstructed from the amino acid sequence of the CP protein of the eight GFLV isolates and ArMV‐S. Figure S3 Evaluation of the resistance of T2 lines 23EG16 9 and 23EG38 4 to infection by viral RNA. [file PBI-16-660-s001.docx]

Supplementary figures

**Figure S1** **Phylogenetic tree of Nanobodies (Nbs) directed against GFLV.** The protein sequence of 23 GFLV Nbs was aligned with the ClustalO program and a tree using the Neighbor-Joining algorithm was generated. Bootstrap values representing the resampling of 100 replicates are indicated above each branch node. The scale bar represents 0.1 change per amino‑acid position determined by Kimura protein distance measure method. The Nbs clustered in 11 different families as highlighted by colored boxes and identified by numbers. Nb23 belongs to family #1. The amino-acid sequence of PVSP6A, a poliovirus neutralizing Nb, was used as out-group.

**Figure S2** **Unrooted phylogenetic tree reconstructed from the amino acid sequence of the CP protein of the eight GFLV isolates and ArMV-S**. The phylogenetic relationships were determined with the Neighbor-Joining method. The percentage of replicate trees in which the associated taxa clustered together in the bootstrap test (1000 replicates) are shown next to the branches.

The GenBank accession numbers of the sequences that were used are as follows: ArMV-S (X81814 for polyprotein P2-U), GHu (EF426852), BE4.11 (MF182352), F13 (NC_003623), BE5.19-C26b (FJ544937), CO2-A17d (AY370959), BUChardT60 (MF182353). The isolate B844 is composed of two RNA2 molecules, RNA2a and RNA2b with accessions numbers MF182354 and MF182355, respectively. The isolate F13TD is a mutant of F13 strain (Schellenberger et al., 2011).

Saitou N. and Nei M. (1987). The neighbor-joining method: A new method for reconstructing phylogenetic trees. Molecular Biology and Evolution 4:406-425.

 Figure S3 Evaluation of the resistance of T2 lines 23EG16‑9 and 23EG38‑4 to infection by viral RNA. EG11‑3, 23EG16‑9 and 23EG38‑4 plants were mechanically inoculated with 360 ng of GFLV‑GHu viral RNA and apical non-inoculated leaves analyzed by DAS‑ELISA at 21 dpi. Each dot corresponds to a single sample and represents the mean relative absorbance at 405 nm of experimental duplicates. Non-inoculated plants were used as negative control (NC). Number of plants tested (n) and percentage of infections (%) are indicated below each column. Note that line 23EG16‑9 is fully resistant to viral RNA whereas only one plant tested positive for GFLV in line 23EG38‑4.
